# Frequency of 16S rRNA Methylase and Aminoglycoside-Modifying Enzyme Genes among Clinical Isolates of *Acinetobacter baumannii* in Iran

**Published:** 2017-10-01

**Authors:** Mehrdad Gholami, Mohammadreza Haghshenas, Mona Moshiri, Shabnam Razavi, Abazar Pournajaf, Gholamreza Irajian, Mohsen Heidary

**Affiliations:** 1 *Dept. of Microbiology, Faculty of Medicine, Iran University of Medical Sciences, Tehran, Iran*; 2 *Microbial Biotechnology Research Center, Iran University of Medical Sciences, Tehran, Iran*; 3 *Dept. of Microbiology, Molecular and Cell-Biology Research Center, Faculty of Medicine, Mazandaran University of Medical Sciences, Sari, Iran*; 4 *Dept. of Pathobiology, Division of Microbiology, Faculty of Public Health, Tehran University of Medical Sciences, Tehran, Iran*; 5 *Dept. of Microbiology, School of Medicine, Shahid Beheshti University of Medical Sciences, Tehran, Iran*

**Keywords:** *Acinetobacter baumannii*, Multidrug resistant, Aminoglycoside-Modifying Enzymes, Iran

## Abstract

**Background & objective:**

Multidrug-resistant *Acinetobacter baumannii* (MDR-AB) is an important nosocomial pathogen which is associated with significant morbidity and mortality, particularly in high-risk populations. Aminoglycoside-modifying enzymes (AMEs) and 16S ribosomal RNA (16S rRNA) methylation are two important mechanisms of resistance to aminoglycosides. The aim of this study was to determine the prevalence of 16S rRNA methylase (*armA*, *rmtA*, *rmtB*, *rmtC*, and *rmtD*), and the AME genes [*aac(6′)-Ib*, *aac(3)-I*, *ant(3′′)-I*, *aph(3′)-I* and *aac(6')-Id*], among clinical isolates of *A. baumannii* in Tehran, Iran.

**Methods:**

Between November 2015 to July 2016, a total of 110 clinical strains of *A. baumannii* were isolated from patients in two teaching hospitals in Tehran, Iran. Antimicrobial susceptibility testing was performed according to Clinical and Laboratory Standards Institute guidelines. The presence of genes encoding the AMEs and 16S rRNA methylases responsible for resistance was investigated by multiplex polymerase chain reaction.

**Results:**

The results showed that colistin was an effective antibiotic and could be used as a last-resort treatment of infections caused by MDR-AB**. **The resistance rate to aminoglycosides were 100%, 96.36% and 90.9% for tobramycin, gentamicin and amikacin, respectively. In this study, AME genes of *aac(6′)-Ib*, *aac(3)-I* and *ant(3′′)-I* were most prevalent among the isolated strains.

**Conclusion:**

Markedly high resistance to tobramycin, gentamicin and amikacin was noted in current study. Our results suggested that modifying enzyme genes in conjunction with methylation of 16S rRNA might contribute to aminoglycoside resistance induced in vivo in *A. baumannii*. Further studies are required to determine the prevalence of the aminoglycoside resistance genes in other hospitals of Iran.

## Introduction

Multidrug-resistant *Acinetobacter baumannii* (MDR-AB) have recently emerged as a life-threatening pathogen which is responsible for a variety of healthcare-associated infections (HAIs) and large epidemics in hospitals (1). Due to MDR-strains are resistant to almost all routinely used antimicrobial agents such as; aminoglycosides, fluoroquinolones, tetracyclines, and cephalosporines, they are becoming a major problem in hospitalized patients in hospital environments all around the world (2, 3, 4). Aminoglycosides are broad-spectrum bactericidal antibiotics which are commonly prescribed for the treatment of infections with Gram-negative bacteria (5, 6). In general, aminoglycosides such as tobramycin, gentamicin and amikacin have extensively been used for ttreatment of *A.baumannii* infections (7, 8). Aminoglycoside-modifying enzymes (AMEs) and 16S ribosomal RNA (16S rRNA) methylation are two important mechanisms for antibiotic inactivation and lead to resistance to multiple aminoglycosides in *A. baumannii* (9, 10). According to their functions, AMEs are generally categorized to three types: aminoglycoside phosphotransferase (APS), aminoglycoside acetyltransferase (AAC), and aminoglycoside nucleotidyltransferase (ANT). All three types of AMEs have been identified in clinical isolates of *Acinetobacter* spp. (10). Furthermore, a high number of genes encoding these enzymes are associated with plasmids and transposons, which help in the rapid spreading of antimicrobial resistance through species boundaries. Several AMEs have been recognized in *Acinetobacter* spp., including variants of phosphotransferases APH(3')-I, APH(3')-II, and APH(3')-VI, the acetyltransferases AAC(3)-I, AAC(3)-II, AAC(3)-III, AAC(6')-I, AAC(6')-II, and AAC(6')-III, and the nucleotidyltransferases  ANT(3-)-I, ANT(4')-I, and ANT(2")-I (11,12,13). The aminoglycoside antibiotics bind to the A-position of 16S rRNA in the 30S ribosomal small subunit and interact with protein synthesis. At present, ten types of 16S rRNA methylase genes *(armA, rmtA, rmtB, rmtC, rmtD, rmtE, rmtF, rmtG, rmtH and npmA) *have been described as another main mechanism of aminoglycoside resistance in *A. baumannii*. Hence, these genes can simply move to other bacteria since their resistance genes are commonly located on small plasmids (14, 15). As well as, the 16S rRNA methyltransferase genes are important factors in the increasing prevalence of aminoglycoside resistance among *A. baumannii *strains, the analysis of the acquisition of these genes by clinical isolates is essential for the treatment and prevention of their infections (15). Additionally, the emergence of AMEs and 16S rRNA methylases among *A. baumannii* strains is a serious global threat for the future of antibacterial chemotherapy. With respect to studies such as, Aliakbarzade et al. (16), Karah et al. (17), and Joshi et al. (18), in this field and also, high dissemination and high prevalence of resistance-related genes, it is important to pay greater attention for choosing the best therapy options for patients and optimizing preventive policies to avoid spreading resistance-encoding genes. Therefore, the aim of this study was to investigate the prevalence of 16S rRNA methylase [*armA*, *rmtA*, *rmtB*, *rmtC*, and *rmtD*], and the AME genes [*aac(6′)-Ib*, *aac(3)-I*, *ant(3′′)-I*, *aph(3′)-I* and *aac(6')-Id*], among clinical isolates of *A. baumannii* from Tehran, Iran.

## Materials and Methods

**Inclusion/exclusion criteria**

All clinical samples which contain *Acinetobacter* isolates, including both gender, and from all age groups including infants to elderly without considering risk factor were involved in this study. Hence, all duplicated clinical isolates were excluded from this study. All isolates harboring mixed microorganisms (which contains *Acinetobacter*) were also excluded.

**Specimen collection and strain identification**

The current study was a descriptive cross-sectional study. A total of 110 consecutive non-duplicate strains of *A. baumannii* were collected from two teaching hospitals between November 2015 to July 2016. These clinical strains were isolated aseptically from wound, tracheal tube, pleural fluid, blood, urine and sputum of hospitalized patients in Milad and Shahid Motahari hospitals, Tehran, Iran. The isolates were identified based on standard bacteriological tests including gram staining, motility, oxidase, methyl red, voges-proskauer, simmon's citrate, urease and grown on MacConkey agar (16). The confirmation was done using the Microgen identification kit (Microgen Bioproducts Co., UK). In addition, all isolates were examined by PCR amplification for the presence of the *bla*OXA-51-like beta-lactamase gene intrinsic to *A. baumannii*, according to previously described method (19), and then the isolates were stored at –70°C in glycerol skim milk broth.

**Antimicrobial susceptibility testing**

Antimicrobial susceptibility was performed using Kirby-Bauer disc diffusion method according to the Clinical and Laboratory Standards Institute (CLSI) guidelines (20). The discs and contents which were used were as follows: tetracycline (TE: 10 μg), meropenem (MEM: 10 μg), amikacin (AK: 30 μg), imipenem (IPM: 10 μg), cefotaxime (CTX: 30 μg), ceftriaxone (CRO: 30 μg), piperacillin/tazobactam (PTZ: 100/10 μg), piperacillin (PIP: 100 μg), ceftazidime (CAZ: 30 μg), ciprofloxacin (CIP: 5 μg), cefepime (FEP: 30 μg), trimethoprim-sulfamethoxazole (SXT: 2.5 μg), gentamicin (GEN: 10 μg) and Tobramycin ( TOB: 10 μg) (All purchased from Mast Group, Merseyside, UK). Briefly, a microbial suspension was obtained from overnight cultures. The turbidity of each bacterial suspension was adjusted equivalent to a turbidity of 0.5 McFarland standard and then inoculated onto Müller-Hinton agar plates (Merck, Darmstadt, Germany). The diameter zone of inhibition (mm) around the discs were measured after incubation at 37ºC for 20 - 24 hours. 

**Minimum Inhibitory Concentration (MIC)**

For colistin, amikacin and gentamicin MIC was determined by the broth microdilution protocol according to the CLSI. Antibiotic powders were dissolved in an appropriate solvent according to the manufacturer’s recommendations. MICs for amikacin/gentamicin ranging from 0.25 to 512 μg/ml and 0.25 to 128 μg/ml for colistin, were tested. Each well of a 96-well microtiter plate (Extra Gene-Company) contained a total volume of 100 μL of the antibiotic dilution and Müller-Hinton broth medium. Then, the 0.5 McFarland suspension was diluted 1:20 to yield 5 × 10^6^ CFU/ml. When 0.01 ml of this suspension was inoculated into the broth, the final test concentration of the bacteria was approximately 5 × 10^5^ CFU/ml. The correct density of the turbidity standard was verified by measuring absorbance using a spectrophotometer. The microtiter plates were incubated at 37°C for 20-24 hours. The MIC was taken as the lowest concentration of the antibiotic which inhibited the growth of the isolates. The MIC_50_ and MIC_90_ of the antibiotics were calculated by MIC that inhibited 50% and 90% of the isolates, respectively. *Escherichia coli *ATCC 25922 carried out as quality control (QC) strain for all suspectibility testing assays.

**DNA extraction**

Genomic DNA was extracted from fresh overnight cultures grown on Luria-Bertani (LB) agar plates (Difco Laboratories, Detroit, Mich.) by the phenol-chloroform method (21). Extracted DNA was resolved in 100 µL of TE buffer (10 mM Tris, 1 mM EDTA [pH 8.0]) plus 10 µL of RNase (Sigma, St. Louis, Mo.) for removal of contaminating RNA. The concentration and quality of the extracted DNA were evaluated using a Nanodrop spectrophotometer (ND-1000; Thermo Scientiﬁc; Wilmington, DE, USA). Purified DNA was aliquoted and preserved at -20°C. 

**Screening for the presence of aminoglycoside resistance genes**

The specific primer sequences sets used in this study are listed in Table 1. 

The Primer Basic Local Alignment Search Tool (NCBI BLAST) software and MFEprimer-2.0 server, a fast thermodynamics-based program, was used for checking PCR primer specificity. To aid recognition in a multiplex format, the length of PCR products were selected so that there was ˷100 bp difference amongst each subgroup. Multiplex-PCR was performed with PCR system (Eppendorf Co., Germany) for detection of AMEs (*aac (6′)-Ib*, *aac (3)-I*, *ant (3′′)-I*, *aph (3′)-I*, *aac (6')-Id*) and 16S rRNA methylases (*armA*, *rmtA*, *rmtB*, *rmtC*, and *rmtD*) genes. The M-PCR I was undertaken in a final volume of 25 µl containing 2.5 µl of 10X PCR buffer (Thermo Fisher Scientific, Inc.), 2 µl MgCl_2_ (0.8 mM), 1.5 µl of mixed dNTP (10 μM), 1.5 µl of each the AMEs primer (10 pmol), 1 µl of DNA Taq polymerase (2 U) (Takara Bio, Inc., Dalian, China), 1 μl of template DNA and sterile distilled water. The nucleic acid amplification was performed with the following conditions: 94˚C for 3 minutes, followed by 30 cycles of 94˚C for 50 seconds, 56˚C for 40 seconds and 72˚C for 1 minute and final extension for 6 minutes. The mix for the detection of 16S rRNA methylase genes contained 2 µl of mixed dNTP (10 mM), 2 μl of 10X PCR buffer, 0.4 µl of DNA Taq polymerase (5 U), 1.5 µl MgCl_2_ (0.8 mM), 1.25 µl of each primer (10 pmol), 1μL of template, DNA and sterile distilled water to a final volume of 25 μl. The M-PCR II assay was performed, using the following parameters: initial denaturation for 1 minute at 95°C, 33 cycles at 94˚C for 45 seconds, 55˚C for 45 seconds, and 72˚C for 1 minute, followed by a final extension during 4 minutes at 72˚C. The PCR products were electrophoresed (Bio Rad, USA) in 1.5% (W/V) agarose gels (SinaClon, Iran) in TAE buffer (0.04 M Tris-acetate, 0.001 M EDTA [pH 8.2]) for 60 minutes at 120 V. Gels were stained with 2 μg/ml ethidium bromide (EtBr) and visualized by irradiation with UV light. Amplicons were identified by estimation of their lengths (in base pairs) using a DNA ladder (Fermentase, CA). Positive and negative controls were included with each reaction.

**Table 1:** The primer sequences used in this study

Reference	Amplicon size (bp)	Primer	Sequence (5′–3′)	Target gene	
22	169	aac(3)-I -F	ACCTACTCCCAACATCAGCC	***aac(3)-I***	***aminoglycoside-modifying enzymes***
		aac(3)-I -R	ATATAGATCTCACTACGCGC		
22	435	aac(6')-Id -F	ATGATTAGAAAAGCAACTGTCCAAG	***aac(6')-Id***	
		aac(6')-Id -R	TTAAAGTTGCTTTGTAAAACAAATC		
22	519	aac(6')-Ib -F	ATGACTGAGCATGACCTTGC	***aac(6')-Ib***	
		aac(6')-Ib -R	TTAGGCATCACTGCGTGTTC		
22	284	ant(3'')-I -F	TGATTTGCTGGTTACGGTGAC	***ant(3'')-I***	
		ant(3'')-I -R	CGCTATGTTCTCTTGCTTTTG		
22	816	aph(3')-I -F	ATGTGCCATATTCAACGGGAAACG	***aph(3')-I***	
		aph(3')-I -R	TCAGAAAAACTCATCGAGCATCAA		
22	774	armA -F	ATGGATAAGAATGATGTTGTTAAG	***armA***	***16S rRNA methylases***
		armA -R	TTATTTCTGAAATCCACTAGTAATTA		
22	315	rmtA -F	CCTAGCGTCCATCCTTTCCTC	***rmtA***	
		rmtA-I -R	AGCGATATCCAACACACGATGG		
23	173	rmtB -F	GCT TTC TGC GGG CGA TGT AA	***rmtB***	
		rmtB -R	ATG CAA TGC CGC GCT CGT AT		
22	846	rmtC -F	ATGAAAACCAACGATAATTATC	***rmtC***	
		rmtC -R	TTACAATCTCGATACGATAAAATAC		
23	401	rmtD -F	CGG CAC GCG ATT GGG AAG C	***rmtD***	
		rmtD -R	CGG AAA CGA TGC GAC GAT		

**Statistical analysis**

The Minitab16 software (Minitab Inc., Minnesota, USA) was used for the statistical analyses. A P-value of <0.05 and 95% confidence intervals were used to determine significant.

**Ethics statement**

Ethical approval was not needed for the survey, since there was no direct patient involvement and only bacterial isolates were retrospectively studied. Additionally, all clinical samples were unidentified and no recognizable patient information was available.

## Results

Overall, 51 (46.36%) and 59 (53.63%) strains were collected from Shahid Motahari and Milad hospitals respectively. All 110 *A. baumanni* isolates had positive results for *bla*_OXA-51-like _gene. These strains were mostly isolated from wound samples (n=51, 46.36%), followed by tracheal tube (n=37, 33.63). The other samples were isolated from pleural fluid (n=8, 7.27%), blood (n=8, 7.27%), urine (n=4, 3.63%), and sputum (n=2, 1.81%). Seventy-one (64.5%) strains were isolated from male patients and 39 (35.45 %) from female patients. The patients infected with MDR-AB ranged in age from 17 to 100 years and the average age of patients was 52 +/- 17 years (Mean +/- SD). In the current study, colistin exhibited good activity against the MDR-AB strains (MIC_50_, 0.5 μg/mL, and MIC_90_, 1 μg/ml). Also, according to antibiotics susceptibility testing by disc diffusion, all isolates of *A. baumannii* were defined as MDR and resistant to almost all available antibiotics (Table 2).

**Table 2:** Antibiotic susceptibility testing results

**Antimicrobial class**	**Antibiotic**	**Resistant** **No (%)**	**Intermediate No (%)**	**Susceptible** **No (%)**
**Aminoglycosides**	Gentamicin	106 (96.36%)	0 (0%)	4 (3.63%)
	Amikacin	100 (90.9%)	5 (4.54%)	5 (4.54%)
	Tobramycin	110 (100%)	0 (0%)	0 (0%)
**Folate pathway inhibitors**	Trimethoprim-sulfamethoxazole	110 (100%)	0 (0%)	0 (0%)
**Carbapenems**	Imipenem	108 (98.18%)	2 (1.81%)	0(0%)
	Meropenem	110(100%)	0 (0%)	0 (0%)
**Cephems**	Cefotaxime	110 (100%)	0 (0%)	0 (0%)
	Ceftriaxone	110 (100%)	0 (0%)	0(0%)
	Cefepime	110(100%)	0 (0%)	0 (0%)
	Ceftazidime	110 (100%)	0 (0%)	0 (0%)
**Fluoroquinolones**	Ciprofloxacin	110 (100%)	0 (0%)	0(0%)
**Tetracyclines**	Tetracycline	95 (86.36%)	12 (10.9%)	3(2.72%)
**β-lactam/β-lactamase inhibitor combinations**	Piperacillin/Tazobactam	110 (100%)	0 (0%)	0 (0%)
**Penicillins**	Piperacillin	110 (100%)	0 (0%)	0(0%)

**Figure 1:**
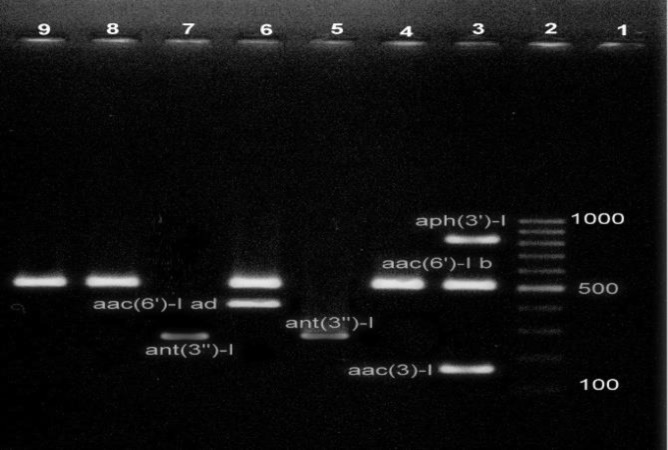
M-PCR I simultaneously amplified *aac(3)-I* (169 bp), *aac(6')-I*d (435 bp), *aac(6')-Ib* (519 bp), *ant(3'')-I* (284) and *aph(3')-I* (816 bp). Lane 1, was the negative control. Lane 2, 100 bp DNA Ladder, Lanes 3-9 amplified products of studied AME genes

**Figure 2:**
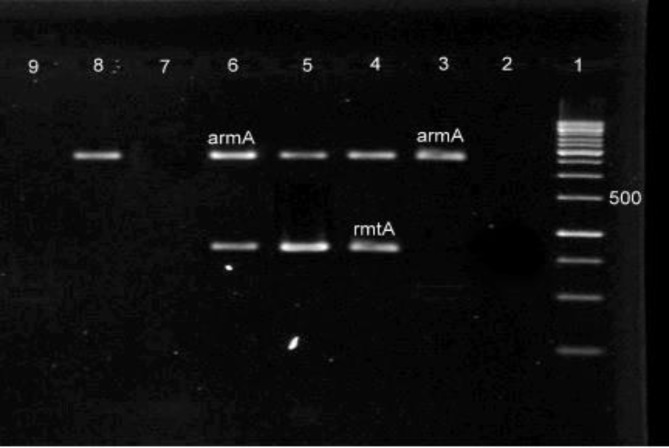
M-PCR II simultaneously amplified *armA* (774 bp) and *rmtA* (315 bp). Lane 1, 100 bp DNA Ladder. Lane 2, negative control. Lanes 3-6 and 8, amplified products of studied 16S rRNA methylase genes, Lanes 7 and 9, had negative results for all 16S rRNA methylase genes

 The *Acinetobacter* isolate was specified as MDR if it was resistant to at least 3 classes of antibiotics (24). The rate of resistance to aminoglycosides was as follows; tobramycin 100%, gentamicin 96.36%, and amikacin 90.9%. The MIC_50_ and MIC_90_ of gentamicin was determined as 128 μg/ml and 512 μg/ml, respectively and MIC_50_ and MIC_90 _of amikacin was determined >128 μg/ml. High MIC values would also predict the limited efficacy of those antibiotics against MDR-AB infections. The frequency of *aac(6′)-Ib*, *aac(3)-I*, *ant(3′′)-I*, *aph(3′)-I* and *aac(6')-Id* genes were 75.45% (83/110), 69.09% (76/110), 57.27% (63/110), 38.18% (42/110) and 1.81% (2/110), respectively. Furthermore, *armA* and *rmtA* genes were detected in 26.36% (29/110) and 2.72% (3/110) of the strains repectively. However, none of the strains carried *rmtB*, *rmtC*, and *rmtD* genes. Figures 1 and 2 show the agarose gel electrophoresis MPCR-amplified products of AMEs and 16S rRNA methylase genes, respectively. Coexistence of aminoglycoside resistance genes among the isolates are shown in Table 3.

**Table 3.** Coexistence of aminoglycoside resistance genes among MDR-AB isolates

Patterns	No. of isolates	Positive rate (%)
*aac(6′)-Ib + aac(3)-I + ant(3′′ )-I*	22	20
*aac(3)-I *+* aac(6′)-Ib *+ *aph(3′)-I*	21	19.09
*aac(6′)-Ib *+* ant(3′′ )- I*	20	18.18
*ant(3′′ )- I* + *armA*+ *aph(3′)-I*	14	12.72
*aac(3)-I* + *armA*	13	11.81
*aac(6′)-Ib *+ *aac(3)-I*	6	5.45
*aac(3)-I* + *aph(3′)-I* +* aac(6′)-Ib*	5	4.54
*aac(6′)-Ib *+ *ant(3′′ )- I *+ * aac(3)-I*	4	3.63
*armA *+ *aac(6’)-Ib *+ *aac(6')-Id*	2	1.81
*aac(3)-I *+* ant(3′′ )- I *+ *aph(3′)-I* +* rmtA*	2	1.81
*aac(6′)-Ib *+ *rmtA *+*ant(3′′ )- I *+ * aac(3)-I*	1	0.9

## Discussion

*A. baumannii* is a ubiquitous opportunistic pathogen that is especially successful at colonizing and persisting in hospital environments (25). Emerging importance of MDR-AB critically ill patients and their capacity to be more resistant to antimicrobial agents necessitates immediate action (4, 25). 

In spite of the emergence of new antibiotics, aminoglycosides are still used in combination with β-lactams for treatment of MDR-AB infections in hospitalized patients (26). The results of the current study showed that all tested isolates were significantly resistant to most accessible treatment options such as gentamicin (96.36%) and amikacin (90.9%). This rate of antibiotic resistance in developing countries has increased significantly compared to developed countries during the recent years (27, 28). This may be due to antibiotic stewardship programs and infection control policies in developed countries. In this study, 100% of the isolates were resistant to tobramycin, trimethoprim-sulfamethoxazole, meropenem, cefotaxime, ceftriaxone, cefepime, ceftazidime, ciprofloxacin, piperacillin/tazobactam and piperacillin. These results were consistent with previous Iranian studies on drug resistance of *A. baumannii* (29, 30), as well as other countries such as China, Turkey and Pakistan (31, 32, 33). As expected, the most effective antibiotic with appreciable activity against the studied isolates was colistin. In agreement with our data, the results of previous studies in Iran (29, 34, 35) have shown that colistin-based therapy for treatment of diseases caused by MDR-AB had an appropriate clinical manifestation and decreased mortality. 

Many factors affect the dissemination of antibiotic resistance genes. Although some of *Acinetobacter* spp., have shown intrinsically resistance to some aminoglycosides, resistance genes have also been found in transposons, plasmids and integrons. The rapid emergence of resistance to aminoglycosides in clinical isolates of *Acinetobacter* has been linked to their ability to acquire these resistance determinants. A wide range of the aminoglycoside resistance genes have been reported in *A. baumannii* (36). Several factors such as geographical regions, misuse of antibiotics, and inappropriate prescribing of aminoglycosides can play a significant role in the prevalence of aminoglycoside resistance genes (37). For instance, in Belgium the *aac(3)-Ia *gene (contribut to the gentamicin and tobramycin resistance) was commonly identified in *Acinetobacter* strains (38). Additionally, it was shown that the distribution of amikacin resistance among *A. baumannii* isolated in Spain was correlated with an epidemic strain harboring the aph(3′)-VIa (conferring resistance to kanamycin and neomycin) gene (39). In the current study, MPCR test was used to determine 16S rRNA methylase and AME encoding genes in clinically relevant A. baumannii isolates. Our study shows that the multiplex PCR method is a fast, reliable, and powerful technique for simultaneous detection of multiple factors associated with aminoglycoside-resistance. For epidemiologic analysis, MPCR method may be very helpful than using a conventional PCR for targeting each gene. The results of this study are consistent with those reported by Dillon et al. (40) and Poirel et al. (41) and show that the MPCR is a suitable and fast diagnostic application for the management and screening of genes that confer resistance to antibiotics. Our findings indicate that the *aac(6′)-Ib*, *aac(3)-I*, *aph(3′)-I,* and *armA *genes are more prevalent than other genes; *aac(6')-Id *and *rmtA *genes were found only at a very low incidence in our tested isolates. In a study conducted by Wen JT et al. in China, the prevalence of genes encoding *aac(3')-I*, *aac(6')-Ib*, *ant(3'')-I*, and *aph(3')-I* were 90%, 90%, 85%, and 35.0%, respectively, although other types of 16S rRNA methylase including *rmtA*, *rmtB*, *rmtC*, *rmtD* and* armA* were not detected among the MDR-AB isolates (22). A study conducted by Xiao et al. on *A. baumannii *isolates showed that the prevalence of *aac(3)-I*, *aac(6ʹ)-Ib*, *ant(2ʺ)-I*, and *aph(3')-I* genes were 10.7%, 17.9%, 14.3%, and 17.9%, respectively, and except *armA* (17.9%), other types of 16S rRNA methylase genes were not detected in any of the isolates (31). Aliakbarzade et al. (16) reported among the 103 *A. baumannii* isolates in Tabriz (Northwest Iran), 65.11 % and 60.46 % were positive for *aacC1 *and *aph(3')-VIa*, respectively. However in another study performed by Moniri et al. in Kashan (Isfahan Province, Iran) the frequency of *aph(3')-VIa*, *aac(3)-Ia*, *ant(3')-Ia* and *ant(2")-Ia* genes were 65%, 63.3%, 41.7%, and 3.3%, respectively (42). The 16S rRNA methylases could confer high-level resistance to aminoglycosides (15). In our study, *armA* was the most prevalent 16S rRNA methylase gene (26.36%), which is in concordance with reports of Vajihe Sheikhalizadeh et al. (44). This gene could be moved to other bacteria by conjugation and conferred high-level resistance to kanamycin, tobramycin, amikacin and gentamicin. These results exhibited that clinical isolates of *Acinetobacter* in different regions, harboring various types of aminoglycoside resistance genes and coexistence of resistance genes could be found in MDR-AB. In conclusion, the current study revealed that high prevalence of aminoglycoside resistance genes among MDR-AB isolates may be associated with AMEs genes, and 16S rRNA methylase genes were not prevalent in the tested isolates. The other mechanisms of resistance to aminoglycosides such as efflux pumps in MDR-AB strains should be considered in further investigations. Finally, paying attention to the recommended guidelines and implementing infection control policies to decrease the rate of MDR strains must be prioritized in therapeutic centers.
